# Synthetic metabolic pathway for the production of 1-alkenes from lignin-derived molecules

**DOI:** 10.1186/s12934-019-1097-x

**Published:** 2019-03-11

**Authors:** Jin Luo, Tapio Lehtinen, Elena Efimova, Ville Santala, Suvi Santala

**Affiliations:** Faculty of Engineering and Natural Sciences, Tampere University, PO Box 527, Tampere, FI-33014 Finland

**Keywords:** Lignin, Ferulate, 1-Alkenes, Adaptive laboratory evolution, *Acinetobacter baylyi*

## Abstract

**Background:**

Integration of synthetic metabolic pathways to catabolically diverse chassis provides new opportunities for sustainable production. One attractive scenario is the use of abundant waste material to produce a readily collectable product, which can reduce the production costs. Towards that end, we established a cellular platform for the production of semivolatile medium-chain α-olefins from lignin-derived molecules: we constructed 1-undecene synthesis pathway in *Acinetobacter baylyi* ADP1 using ferulate, a lignin-derived model compound, as the sole carbon source for both cell growth and product synthesis.

**Results:**

In order to overcome the toxicity of ferulate, we first applied adaptive laboratory evolution to *A. baylyi* ADP1, resulting in a highly ferulate-tolerant strain. The adapted strain exhibited robust growth in 100 mM ferulate while the growth of the wild type strain was completely inhibited. Next, we expressed two heterologous enzymes in the wild type strain to confer 1-undecene production from glucose: a fatty acid decarboxylase UndA from *Pseudomonas putida*, and a thioesterase ‘TesA from *Escherichia coli*. Finally, we constructed the 1-undecene synthesis pathway in the ferulate-tolerant strain. The engineered cells were able to produce biomass and 1-undecene solely from ferulate, and excreted the product directly to the culture headspace.

**Conclusions:**

In this study, we employed a bacterium *Acinetobacter baylyi* ADP1 to integrate a natural aromatics degrading pathway to a synthetic production route, allowing the upgradation of lignin derived molecules to value-added products. We developed a highly ferulate-tolerant strain and established the biosynthesis of an industrially relevant chemical, 1-undecene, solely from the lignin-derived model compound. This study reports the production of alkenes from lignin derived molecules for the first time and demonstrates the potential of lignin as a sustainable resource in the bio-based synthesis of valuable products.

**Electronic supplementary material:**

The online version of this article (10.1186/s12934-019-1097-x) contains supplementary material, which is available to authorized users.

## Background

The concerns of energy security and environmental issues are driving the development of sustainable and environment-friendly processes for the production of chemicals and fuels. To that end, lignocellulose biorefining has gained substantial attention as a solution to mitigate the dependence on petroleum-based industry [1]. Lignocellulose is the most abundant biopolymer on Earth, holding huge potential as the feedstock for sustainable bioproduction. However, efficient use of lignocellulose is somewhat hindered as lignin, a major component of lignocellulose, is underutilized due to its recalcitrance and inherent heterogeneity. Along with the development of second generation biorefineries, an increasing amount of lignin will be generated as a by-product [2]. In addition, pulp and paper industry produce large quantities of lignin-rich waste as a by-product [2, 3]. Thus, it is of high priority to develop technologies for lignin valorization with respect to economic efficiency and environmental sustainability.

To boost the value of lignin, strategies have been developed for lignin depolymerization and subsequent valorization [4]. However, lignin depolymerization yields heterogeneous and recalcitrant aromatic molecules, which complicates the downstream processing. Nevertheless, some microorganisms have evolved the pathways for aromatic catabolism [5–7], which provides the opportunity to convert these lignin-derived molecules (LDMs) into different specialized end-products. *Acinetobacter baylyi* ADP1 is one of the microorganisms that has been reported being able to catabolize various LDMs and even directly depolymerize lignin [8, 9]. A “funneling pathway” is employed by *A. baylyi* ADP1 to convert various aromatic molecules into central intermediates, such as protocatechuate and catechol [6, 10, 11]. The central intermediates are further catabolized through β-ketoadipate pathway, resulting in ring-opened compounds that will be directed to central metabolism. Moreover, *A. baylyi* ADP1 is readily genetically engineerable due to its natural transformability and recombination ability [12] and has been shown to produce a variety of industrially relevant compounds, such as wax esters, long chain alkanes and triacylglycerol, by native and non-native pathways [13–16]. Thus, *A. baylyi* ADP1 could be a potential candidate for lignin valorization.

LDMs are known to be toxic to microorganisms [17–19], which also hinders their use as substrates. In order to develop a tolerant strain, adaptive laboratory evolution (ALE) can be employed. In ALE, microorganisms are successively cultivated under rationally designed selection pressure, e.g. elevated concentrations of inhibitors [20]. The approach can lead to the strain with beneficial changes allowing them to adapt to the stressful condition. ALE has been successfully applied on various microorganisms to improve their tolerance against inhibitory compounds [19, 21, 22].

Bio-based production of hydrocarbons, such as alkanes and alkenes, is of great interest due to their use as advanced “drop-in” biofuels and various fine chemicals [23–26]. In nature, some organisms have been found to possess the pathways for the synthesis of medium-chain (C8–C12) or long-chain (> C12) hydrocarbons [23, 27]. Particularly, medium-chain α-olefins, such as 1-undecene (C11), are attractive molecules for their broad use in detergents, plasticizers and monomers for elastomers [23]. In addition, the molecules can accumulate extracellularly and are semivolatile, which allows them to be collected directly from the culture vessel without cell harvesting and/or product extraction, significantly reducing the costs and labour of downstream processing.

**Fig. 1:**
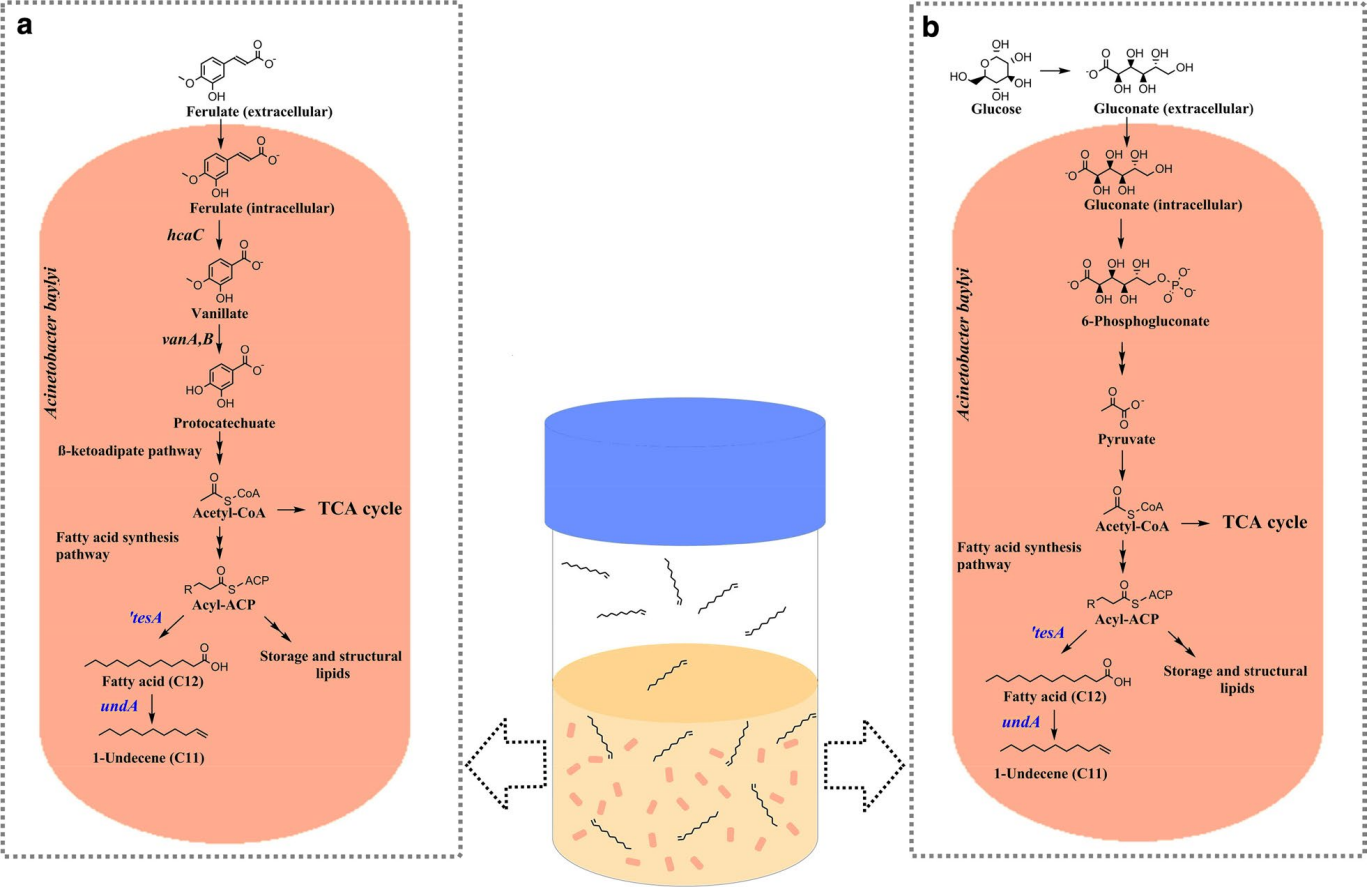
A schematic representation of medium-chain alkene production from ferulate (**a**) and glucose (**b**) by *A. baylyi* ADP1. In *A. baylyi* ADP1, genes hcaC and *vanA,B* encode for coenzyme A ligase and vanillate demethylation machinery respectively, responsible for conversion of ferulate to protocatechuate. Protocatechuate is further converted to the central metabolite, acetyl-CoA (coenzyme A), via β-ketoadipate pathway. Acetyl-CoA is also the precursor of fatty acid synthesis. Genes ‘*tesA* and *undA,* encoding for thioesterase and decarboxylase, respectively, are heterologously expressed in *A. baylyi* ADP1 for 1-undecene production. 1-Undecene is a semivolatile hydrocarbon, which can be directly collected from the culture headspace

Diversion of the natural fatty acid metabolism in microorganisms through a synthetic pathway provides the mean for 1-undecene production (Fig. 1). Recently, a gene *undA* originated from *Pseudomonas* has been discovered to be responsible for 1-undecene (C11) biosynthesis [28]. UndA is an oxygen-activating, nonheme iron (II)-dependent decarboxylase that converts free fatty acids (FFAs) with chain lengths from 10 to 14 to the corresponding terminal alkenes. Thus, the availability of free fatty acids is essential for alkene production. Overproduction of free fatty acids can be potentially realized by expression a cytoplasmic thioesterase that hydrolyzes fatty acyl-ACP (acyl carrier protein), the product of fatty acid synthesis pathway, into free fatty acids. An *Escherichia coli* thioesterase, ‘TesA (a leaderless version of TesA that is targeted in cytosol), has been widely studied and established in free fatty acid overproduction [24, 29]. Other possible alkene synthesis pathways include polyketide synthase pathway and head-to-head hydrocarbon synthesis pathway, in which multiple enzymes and reactions are involved [30, 31]. In comparison, the one-step decarboxylation of FFAs catalyzed by UndA is simpler and highly specific for the production of 1-undecene [32]. Medium chain 1-alkenes have been heterologously synthesized by traditional microbial platforms such as *E. coli* and *Saccharomyces cerevisiae* [28, 33, 34]. However, none of these microorganisms are able to utilize aromatic compounds as substrates for bioproduction. Integration of synthetic pathways to a natural aromatic catabolizing microorganism provides an alternative way for the upgrading of abundantly available LDMs.

In this study, we employed *A. baylyi* ADP1 for the production of 1-undecene from a lignin-derived model compound, ferulate. We applied ALE to improve the tolerance of *A. baylyi* ADP1 against ferulate and introduced the one-step fatty acid decarboxylation pathway in the resulting strain. Finally, we established a synthetic pathway for the direct conversion of LDMs to 1-undecene (Fig. 1). We demonstrate the potential of catabolically diverse bacteria for the synthesis of industrially relevant compounds from an abundant and sustainable substrate.

## Results and discussion

### Adaptation of *A. baylyi* ADP1 to high concentration of ferulate

**Fig. 2:**
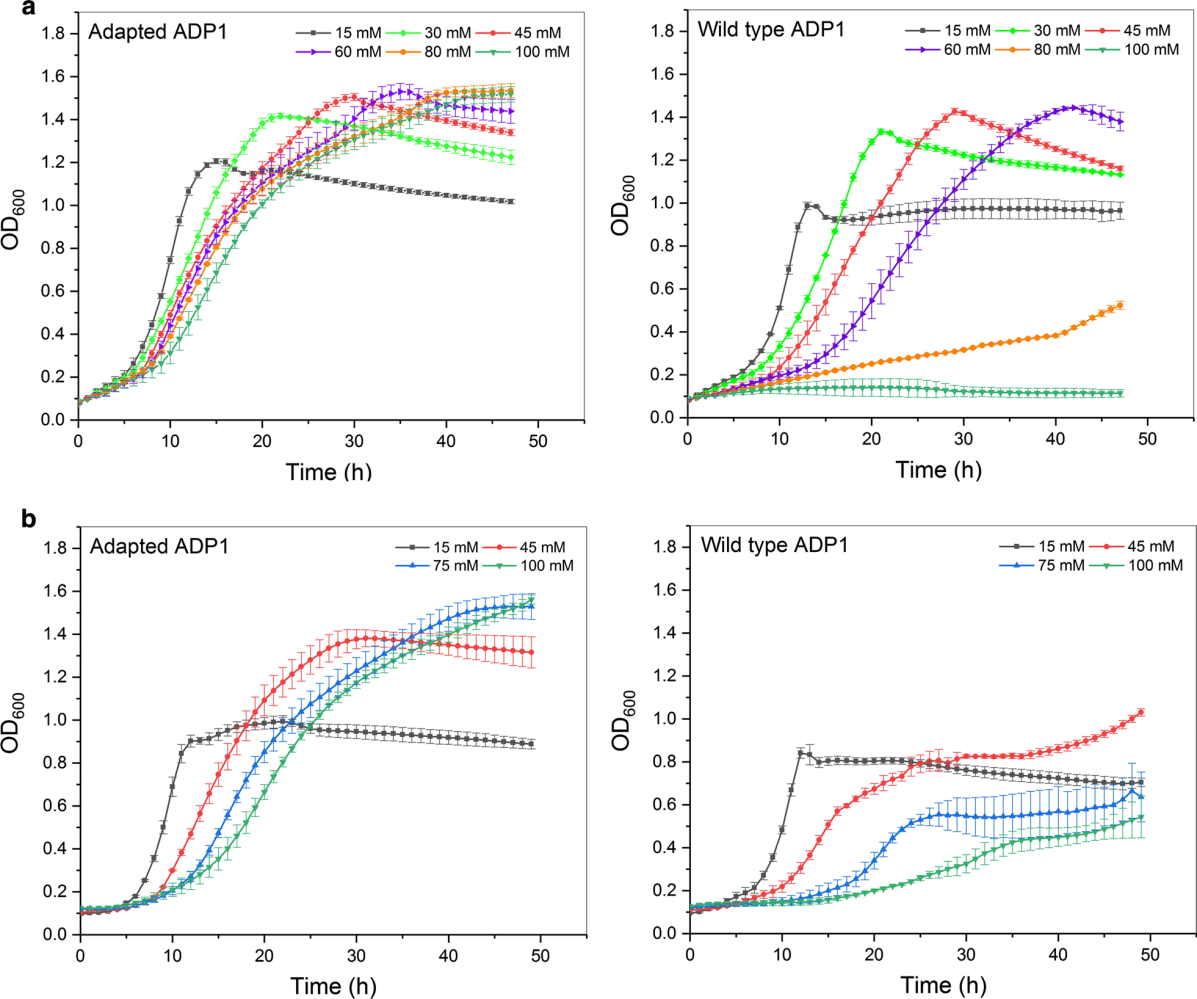
The growth of adapted and wild-type ADP1 on ferulate (**a**) and vanillate (**b**). Adapted and wild type ADP1 were cultured in mineral salts medium supplemented with different concentrations of ferulate and vanillate as sole carbon sources. The mean values and error bars (representing the standard deviations) from three parallel cultures are shown

In this study, ferulate was used as the model compound of LDMs. Ferulic acid is an important building block during lignin biosynthesis [35]. Its salt form, ferulate, is one of the major LDMs that can be obtained from alkaline pretreated lignin [36, 37]. In addition, ferulate is the model compound representing the guaiacyl unit of lignin, which accounts for more than 90% of the total lignin structure units of softwood [4]. The degradation of ferulate by *Acinetobacter* has been previously described [10, 38]. Ferulate is metabolized through vanillate to the central intermediate, protocatechuate. Although *A. baylyi* ADP1 can utilize ferulate as a sole carbon source, it was found out here that the growth rate is reduced or completely inhibited at concentrations above 80 mM (Fig. 2a), which might set limitations to bioprocessing. The mechanism of the inhibition caused by ferulate has not been characterized in detail, but a general mechanism of the inhibition caused by phenolic compounds is thought to be related to their hydrophobicity [17, 39]; phenolic compounds can target cell membranes and interact with lipids and membrane-embedded proteins, breaking the integrity of the membranes. In order to allow the use of LDMs as a substrate for both cell growth and product synthesis, it is a prerequisite to overcome the toxicity of these compounds to host cells. To this end, ALE was employed here for *A. baylyi* ADP1 to improve the tolerance and growth on ferulate. Two populations, from the same parental strain, were concurrently evolved in mineral salts medium containing ferulate as the sole carbon source. The evolution was performed for 2 months, corresponding to 362 and 374 generations for population 1 and population 2, respectively. The initial concentration of ferulate was 45 mM and gradually increased to 125 mM by the end of the evolution. After the evolution, three isolates from each of the two evolved populations were taken for growth comparison. The isolates from the same population showed similar growth profile. The isolates from population 1 showed better and more consistent growth in ferulate than those from population 2. One of the isolates from population 1, designated as adapted ADP1, was selected for further comparison with wild type ADP1 (parental strain). In adapted ADP1, colony morphology did not change based on the observation on agar plates. In addition, the natural transformability was maintained after the ALE.

### Comparison of growth between wild type and adapted ADP1

To compare the growth between the wild type and adapted ADP1 when ferulate was used a sole carbon source, both strains were precultivated in mineral salts medium supplemented with 15 mM ferulate, after which cells were transferred to fresh mediums supplemented with different concentrations of ferulate. Generally, adapted ADP1 showed improved growth compared to the wild-type strain (Fig. 2a). Increase of ferulate concentration from 15 mM to 100 mM negatively influenced the growths of both adapted and wild type ADP1, but the influence was much smaller on the growth of adapted ADP1 than that of the wild type strain. Wild type ADP1 showed poor growth in 80 mM ferulate and the growth was completely inhibited when ferulate concentration was 100 mM. In contrast, adapted ADP1 still exhibited prominent growth in 100 mM ferulate (Fig. 2a). In low ferulate concentration (15 mM – 60 mM) at which both strains could grow well, adapted ADP1 also exhibited faster growth and reached higher optical density (OD) than the wild type strain (Additional file 1: Figure S2).

In the aromatic catabolizing pathway of ADP1, ferulate is first converted into vanillate [10]. Thus, the growth comparison was also performed using vanillate (15 mM, 45 mM, 75 mM and 100 mM) as a sole carbon source. Similarly, adapted ADP1 showed advantage over wild type in terms of growth on vanillate (Fig. 2b); Wild type showed slightly slower growth and lower final OD than the adapted ADP1 already in 15 mM vanillate (Additional file 1: Figure S3). As the concentration was increased, the growth of wild type ADP1 was inhibited to a larger extent. A two-phase growth was observed in the wild type strain when vanillate concentration was more than 45 mM (Fig. 2b). In comparison, adapted ADP1 exhibited similar growth profile as when grown on ferulate. Interestingly, adapted ADP1 did not show significantly improved tolerance against *p*-coumarate, another LDM that is also catabolized by ADP1 via β-ketoadipate pathway (data not shown). Due to the prominent growth of adapted ADP1 in concentration as high as 100 mM, further cultivations were performed with ferulate and vanillate concentrations ranging from 120 mM to 180 mM. Impressively, adapted ADP1 still grew robustly in 180 mM ferulate (Additional file 1: Figure S4 A). The growth on vanillate was inhibited to a large degree when the concentration was higher than 160 mM (Additional file 1: Figure S4 B).

As a result of ALE, the growth from both ferulate and vanillate was significantly improved. The adapted strain showed not only robust growth in high ferulate or vanillate concentration while the growth of wild type was completely inhibited, but also more prominent growth than wild type in low ferulate concentrations.

In a previous study, the genes involved in the tolerance towards coumaric acid, another lignin-derived phenolic compound, were identified in *Pseudomonas putida* [40]. Most of the identified genes were related to membrane stability, transport system, and membrane proteins while the genes involved in the degradation of the compounds only play minor roles in the tolerance [40]. Those identified genes seem to be more related to global stress handling. Here, it seems that the improved tolerance is resulted from a different mechanism, since the adapted strain shows improved tolerance specific to ferulate and vanillate but not to coumarate. The additional methoxyl group on the benzene ring of ferulate and vanillate might play an important role in the mechanism. In addition, the two-phase growth observed in the wild type strain grown on higher vanillate concentration (45 mM–100 mM) might indicate some regulations in the vanillate catabolism. It was reported in *A. baylyi* ADP1 that the downstream metabolite protocatechuate can repress *vanA,B* operon which is responsible for its generation from vanillate, and the repressive effect is increased as protocatechuate concentration increases [41]. It might be possible that high vanillate concentration caused protocatechuate accumulation, leading to repression of vanillate catabolism, which resulted in a two-phase growth. The two-phase growth was not observed in adapted ADP1, indicating that, in addition to the improved tolerance, changes related to the regulation of aromatic catabolism might also occur during the evolution. The correlations between the improved phenotype and the genotype remain to be investigated in future.

### Constructing the synthesis pathway for 1-undecene production

Alkenes represent industrially relevant platform chemicals used in a broad range of applications and products. Interestingly, the short and medium chain molecules are semivolatile, potentially allowing straight-forward collection and continuous production processes. For example, in a continuous cultivation in bioreactor, the volatile 1-undecene could be continuously removed by gas stripping and collected using solvents at the gas outlet. The significance of direct product collection increases when industrial heterogeneous streams with varying composition are used as the feedstock. As a proof-of-concept, our aim was to demonstrate the production of volatile 1-undecene from ferulate without downstream processing.

1-Alkenes have been previously produced in traditional microbial cell factories, such as *E. coli* and *S. cerevisiae*, using either rich medium or minimal medium containing glucose [28, 33, 42, 43]. Chen et al. expressed another decarboxylase OleT in *S. cerevisiae* and increased the production of total intracellular alkenes 67.4-fold to 3.7 mg/l by combinatorial metabolic engineering strategies and process optimization [42]. Liu et al. expressed OleT in FFAs overproducing *E. coli* and obtained 97.6 mg/l of total intra- and extracellular alkenes [43]. However, due to the wide substrate spectrum of OleT (C12 to C20), mixtures of alkenes were produced, long chain alkenes (C15 to C19) being the dominant products [42, 43]. Compared to OleT, UndA has a narrower substrate spectrum towards medium chain length fatty acids. UndA has a hydrophobic substrate-binding pocket extending from the surface of the enzyme to the center [28]. The depth of the pocket limits the chain length of the substrates that can be accepted, rendering UndA preferable to medium chain fatty acids (C10 to C14) [28]. Rui et al. overexpressed a UndA homolog in *E. coli* and obtained 6 mg/l extracellular 1-undecene production [28]. UndB, a membrane-bound desaturase-like enzyme, was later found to be the most efficient among UndA and OleT for converting lauric acid (C12) to 1-undecene [33]. However, UndB accepts free fatty acids ranging from C6 to C18 as substrates [33]. Co-expression of an UndB homolog and UcFatB2, a C12-specific thioesterase for lauric acid synthesis, conferred specific 1-undecene production with a titer of ∼ 55 mg/l in *E. coli* [33]. However, given that UndB is a membrane-bound enzyme, its expression might have a negative effect on cell growth [34]. Due to the substrate preference and cytosolic (and thus potentially less toxic) nature of UndA, it was chosen here for the establishment of the alkene synthesis pathway in *A. baylyi* ADP1.

To confer 1-undecene production, *undA* and *‘tesA* were heterologously expressed in *A. baylyi* ADP1. The thioesterase ‘TesA is responsible for the conversion of acyl-ACPs to free fatty acids, which are the precursors for alkene synthesis. Although ‘TesA prefers C14 acyl-ACPs as the substrate, it accepts acyl-ACPs with chain lengths ranging from 8 to 18 [29]. We therefore hypothesized that the expression of ‘TesA might benefit the synthesis of 1-undecene by increasing the pool of its precursor. For the tunable expression, a cyclohexanone-inducible promoter ChnR/P_chnB_ originally isolated from *A. johnsonii* was used [44]. The expression system has been previously characterized in *E. coli* and *P. putida* [45]. For testing the functionality of the expression system in *A. baylyi*, a plasmid pBAV1C-chn-GFP was constructed. Induction factor of 52 was obtained for 1 mM cyclohexanone after 3 h of induction, indicating strong expression and high signal/noise ratio.

To allow sufficiently controlled expression, plasmid pBAV1C-chn was constructed and used as the vector for the expression of *undA* and ‘*tesA*. Three different plasmids were constructed expressing either UndA, ‘TesA, or both UndA and ‘TesA: pBAV1C-chn-*undA*, pBAV1C-chn-‘*tesA*, and pBAV1C-chn-‘*tesA*-*undA* (Additional file 1: Figure S1). *A. baylyi* ADP1 was then transformed with pBAV1C-chn (empty plasmid control) and the three expression plasmids, designated as ADP1-empty plasmid, ADP1 *UndA*, ADP1 ‘*tesA*, and ADP1 ‘*tesA*-*undA*.

**Fig. 3:**
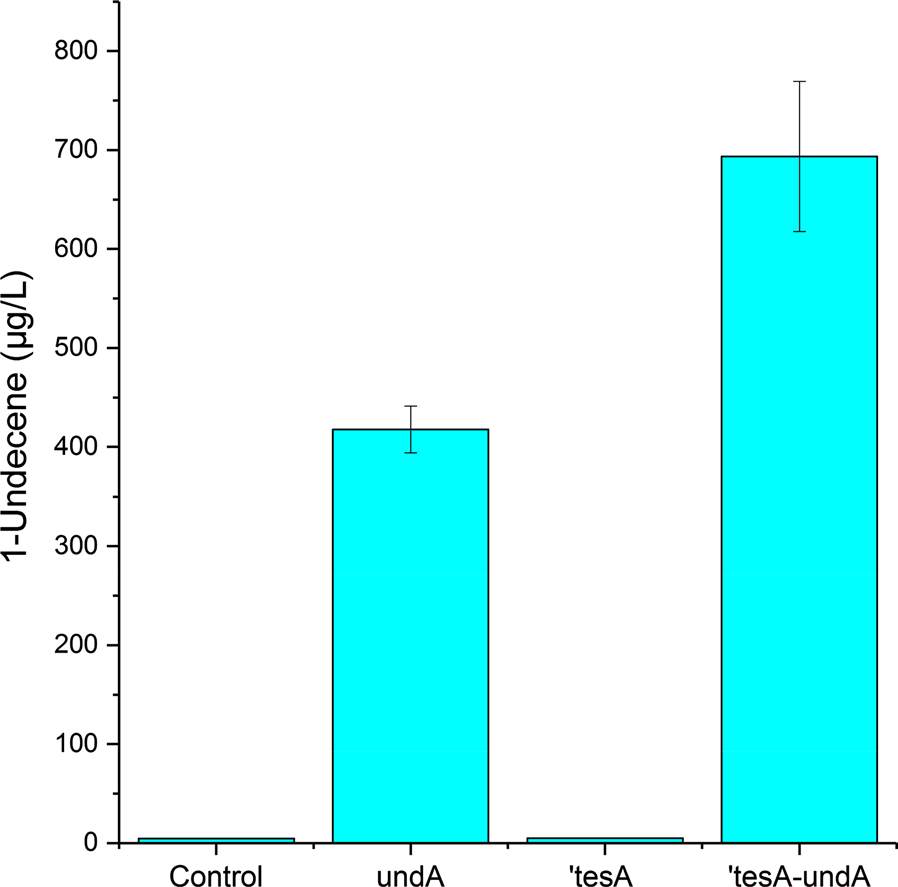
Production of 1-undecene from glucose by *A. baylyi* ADP1 with different constructs. In the histogram, from the left to the right are 1-undecene productions with ADP1- empty plasmid (pBAV1C- chn as control), ADP1 *UndA*, ADP1 ‘*tesA*, and ADP1 ‘*tesA*-*undA*. The mean values and error bars from two parallel cultures are shown

To select the optimal construct, we compared the production of 1-undecene between with the different plasmids, using glucose as the carbon source. The transformants were first cultivated in aerobic condition and induced when the OD reached 1. After 1 h of induction, the cells were transferred to sealed vials and incubated overnight. Although incubation in sealed vials containing limited oxygen negatively influence the cell growth, it allows the accumulation of alkenes in the culture headspace. The production of 1-undecene was directly measured from the headspace of the cultivation vials using solid phase micro-extraction (SPME)–gas chromatography mass spectrometry (GCMS), as described by Rui et al. [28]. The production of 1-undecene by different strains is shown in Fig. 3. ADP1-empty plasmid (control) produced traces of 1-undecene (4.46 ± 0.07 µg/l), indicating the existence of natural 1-undecene synthesis mechanism in ADP1. Either native or non-native alkene synthesis in *A. baylyi* has not been reported before, but UndA homologs have been found in *Acinetobacter* spp. [28], potentially explaining the natural alkene synthesis in ADP1. The production was greatly increased by expressing UndA alone in ADP1 (418 ± 24 µg/l, 93-fold more than the control strain), indicating the availability of FFAs (C12) in ADP1 for 1-undecene synthesis. Because free fatty acids with chain lengths from 10 to 14 typically serve as the substrates for the synthesis of corresponding 1-alkenes by UndA, fatty acyl-CoA and fatty acyl-ACPs are unlikely to be converted by UndA [28]. In microorganisms, the product of fatty acid biosynthesis is fatty acyl-ACP. In *A. baylyi* ADP1, the carbon chain of fatty acyl-ACPs can be elongated up to 18 carbons [14]. The availability of FFAs (C12) can be explained by the existence of a natural intracellular thioesterase in ADP1, which can produce FFAs (C6 to C18) from acyl-ACPs [46]. On the contrary, expressing ‘TesA alone did not have a great influence on 1-undecene production (5.06 ± 0.33 µg/l). The aforementioned results indicate that, in wild type *A. baylyi* ADP1, FFAs decarboxylation is the limiting step for 1-undecene production. ADP1 ‘*tesA*-*undA* had the highest production (694 ± 76 µg/l), being 1.7 fold higher than with ADP1 *undA,* indicating that ‘TesA participated in the conversion of acyl-ACPs to FFAs of suitable chain length, providing more substrates for UndA. However, balancing the expression levels of ‘TesA and UndA could further enhance the production of 1-undecene. In addition to 1-undecene, 1-tridecene was also detected when both ‘TesA and UndA were expressed (Additional file 1: Figure S6), which is reasonable as UndA also accepts C14 fatty acid, the precursor of 1-tridecene, as substrate [28]. The concentration of 1-tridecene was much lower than that of 1-undecene (data not shown), indicating high specificity of UndA towards 1-undecene synthesis. At the end of the cultivation, ADP1 with empty plasmid and ADP1 *undA* reached the OD of more than 2 while the other strains had the OD approximately 1.5 (Additional file 1: Figure S5).

Compared to the previous study [28], in which UndA was used for 1-undecene production, the titer obtained here was lower. This might be due to the difference of metabolism between *E. coli* and *A. baylyi*. In the aforementioned study, *E. coli* was first cultivated under aerobic condition, followed by cultivation in a sealed vial for 1-undecene accumulation [28]. Since *E. coli* is facultative anaerobic, its growth will continue after the oxygen is depleted. Here, *A. baylyi*, an obligate aerobe, was used, and the growth would stop once the oxygen in the sealed vial was depleted. In addition, as the goal was to demonstrate the direct production and collection of 1-undecene without downstream processing, only the alkenes that were secreted by the cells were analyzed. In order to improve the production, the culture set-up should be further developed and optimized; in the current system, the cell growth and consequently 1-undecene production rapidly cease due to the oxygen limitation in sealed vials. For example, cultivations in a bioreactor enabling continuous culture and product recovery would likely significantly improve the productivity. In addition, the current 1-undecene synthesis pathway was not optimized. Employing molecular level strategies, such as increasing the availability of FFAs and co-factors, selection of more efficient enzymes specific to medium-chain length substrate, blocking of competing pathways and balancing the enzyme activities could further improve the production [32, 42, 47].

### 1-Undecene production from ferulate with adapted ADP1

It was demonstrated that the highest 1-undecene production was obtained with pBAV1C-chn-‘*tesA*-*undA* among the constructed plasmids. Thus, adapted ADP1 was transformed with the construct and designated as adapted ADP1 ‘*tesA*-*undA*. For 1-undecene production, both adapted ADP1 ‘*tesA*-*undA* and ADP1 ‘*tesA*-*undA* were precultivated in mineral salts medium supplemented with 5 mM ferulate, followed by batch cultivations in 110 mM ferulate. During the cultivation, biomass and ferulate concentration were monitored. The detection of 1-undecene was carried out by SPME-GCMS.

**Fig. 4:**
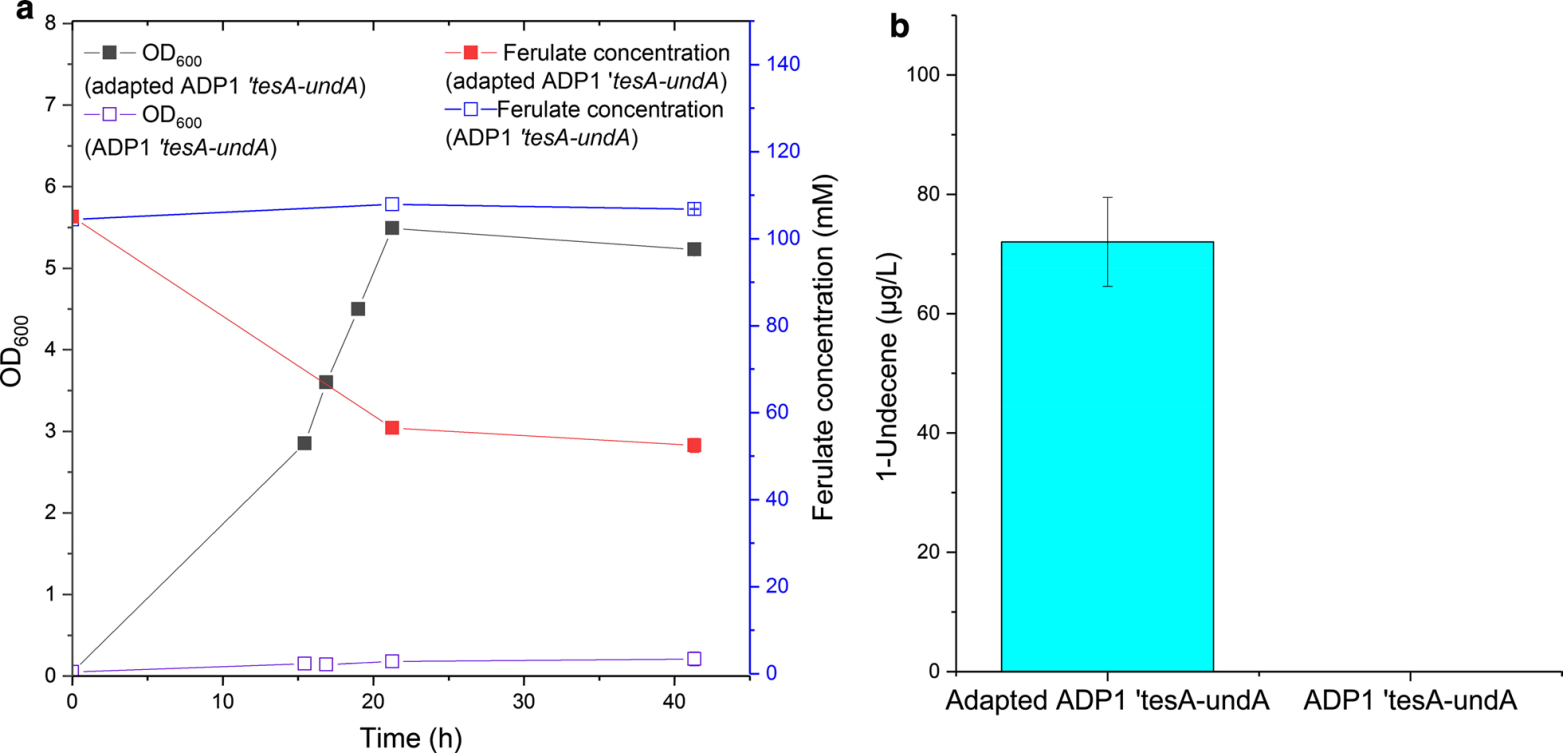
Growth and 1-undecene production by ADP1 *‘tesA*-*undA* and adapted ADP1 ‘*tesA*-*undA* from ferulate. **a** The biomasses (OD) and ferulate concentrations of adapted ADP1 ‘*tesA*-*undA* and ADP1 ‘*tesA*-*undA.* The strains were cultivated in mineral salts medium supplemented with 110 mM ferulate. The cells were induced after 17 h of incubation in aerated flasks, and thereafter incubated in sealed vials for 20 h. The values of the last sampling point are the mean values from two parallel cultures and the error bars represent the standard deviations (the error bars are not visible in the figure). **b** 1-Undecene production using ferulate as the sole carbon source. The samples for 1-undecene measurement were directly collected from the culture headspace. The mean values and error bars from two parallel cultures are shown

As expected, adapted ADP1 ‘*tesA*-*undA* showed distinct advantage over ADP1 ‘*tesA*-*undA* when cultivated in approximately 110 mM ferulate (Fig. 4a). ADP1 ‘*tesA*-*undA* showed almost no growth or ferulate consumption during the cultivation, the final OD being 0.21. On the contrary, adapted ADP1 ‘*tesA*-*undA* showed efficient growth in approximately 110 mM ferulate; the OD was 3.6 at the time of induction, and 5.5 after the following 4.5 h of incubation with the inducer. At this time-point, approximately 58 mM ferulate was consumed. Considering that cultivation in aerobic condition for longer time until all ferulate was consumed may cause loss of 1-undecene due to its volatility, the cultures were immediately transferred to sealed vials for 1-undecene collection. During the incubation sealed vials, due to the oxygen limitation, the OD did not change greatly and only a small amount of additional ferulate was consumed. At the end of incubation, 72 ± 7.5 µg/l (with a yield of 7.2 ± 0.96 µg/g ferulic acid) 1-undecene was produced by adapted ADP1 ‘*tesA*-*undA* while no 1-undecene was produced by ADP1 ‘*tesA*-*undA* (Fig. 4b). The yield was calculated based on the overall ferulate consumption during the whole cultivation, but the production of 1-undecene was induced when the OD was already high and considerable amount of ferulate had been consumed at that point. Cultivation with ferulate as substrate is highly oxygen-consuming, because extra oxygen is needed for the cleavage of aromatic ring for subsequent catabolism [7]. Thus, most of the 1-undencene might be produced during the 4.5 h of induction in the flask but not during the incubation with sealed vials in which there was limited amount of oxygen. Here, adapted ADP1 ‘*tesA*-*undA* was able to produce 1-undecene when cultivated in high concentration of ferulate. In addition, all the required energy and carbon for both generating the catalyst (biomass) and the production of 1-undecene was obtained from ferulate, emphasizing the potential of the used cell platform. Nevertheless, there is still a huge potential to improve the titer and yield by both metabolic engineering strategies and culture set-up optimization.

Although lignin has a great potential as a substrate for bio-based production, one practical problem with its utilization is that the typically employed production hosts cannot degrade and further metabolize the aromatic compounds that are the constituents of lignin [48]. Furthermore, the aromatic compounds are strong growth inhibitors [17]. The current study showed the advantage of *A. baylyi* ADP1 as a novel bacterial production platform for lignin valorization due to its ability of aromatic compound utilization, adaptability, and the possibility to funnel the intermediates to products of interest. However, to realize the upgrading of lignin, many factors should be considered. For example, efficient substrate conversion is the key factor for high productivity. Technologies for lignin depolymerization, such as alkaline pretreatment of lignin, yield low molecular weight LDMs which can serve as the substrates for microbial conversion [4]. However, considering that lignin depolymerization gives rise to a highly heterogeneous mixture of different acids and phenolic compounds, it is necessary to evaluate the utilization of mixed LDMs and even real depolymerization products. It has been reported that the catabolism of aromatic compounds via β-ketoadipate pathway is affected by different regulatory mechanisms, such as catabolite repression, cross regulation and vertical regulation [10, 41]. In order to allow efficient utilization of mixed substrates, further engineering should be employed to reduce the regulatory repression, e.g., by deletion of regulatory elements [37]. In addition, improvement of the tolerance against mixed LDMs is also crucial for obtaining high substrate conversion rate.

## Conclusions

In this study, our aim was to demonstrate the potential of lignin-derived molecules as substrates for the bioproduction of industrially relevant compounds. To that end, we established the production of α-olefins (namely 1-undecene) by a synthetic pathway in *A. baylyi* ADP1 using ferulate, a model compound for lignin monomer, as the sole carbon source. The growth of *A. baylyi* ADP1 from ferulate was significantly improved by ALE, which allowed the use of high (110 mM) ferulate concentration for the production. As a proof-of-concept, we show for the first time that ferulate alone can support both the biomass production and the synthesis of non-native long-chain hydrocarbon product resulting from a multi-step pathway. Our study emphasizes the importance of host selection, and promotes the use of *A. baylyi* ADP1 as a potential chassis for lignin valorization.

## Methods

### Strains

*Escherichia coli* XL1-Blue (Stratagene, USA) was used for plasmid construction and amplification. Wild type *A. baylyi* ADP1 (DSM 24193) was used as the parental strain in ALE to develop adapted ADP1. Wild type *A. baylyi* ADP1 was transformed with plasmid pBAV1C-chn, pBAV1C-chn-*undA*, pBAV1C-chn-*‘tesA*, pBAV1C-chn-*undA*-*‘tesA* and pBAV1C-chn-*‘tesA*-*undA*, designated as ADP1 empty plasmid, ADP1 *undA*, ADP1 ‘*tesA*, ADP1 *undA*-‘*tesA* and ADP1 ‘*tesA*-*undA,* respectively. ADP1 empty plasmid was used as control. The adapted ADP1 was transformed with pBAV1C-chn-*‘tesA*-*undA* and designated as adapted ADP1 ‘*tesA*-*undA*.

### Media

Modified LB medium (10 g/l tryptone, 5 g/l yeast extract, 1 g/l NaCl) was used for plasmid construction and transformation of wild type *A. baylyi* ADP1. The medium was supplemented with 1% glucose as carbon source and 25 μg/ml chloramphenicol as antibiotic when needed. For solid medium, 15 g/l agar was added.

Minimal salts medium (MA/9) was used for the cultivation of plasmid selection. The composition was as follow:Na_2_HPO_4_ 4.40 g/l, KH_2_PO_4_ 3.40 g/l, NH_4_Cl 1.00 g/l, nitrilotriacetic acid 0.008 g/l, NaCl 1.00 g/l, MgSO_4_ 240.70 mg/l, CaCl_2_ 11.10 mg/l, FeCl_3_ 0.50 mg/l. The medium was supplemented with 0.2% casein amino acid, 5% glucose and 25 μg/ml chloramphenicol when indicated.

Mineral salts medium as described by Hartmans et al. [49] was used for the ALE cultivation, the growth comparison in ferulate (wild type ADP1 vs. adapted ADP1), the transformation of adapted ADP1 and the cultivation of 1-undecene production from ferulate. This medium was applied in the experiments related to adapted ADP1 to maintain its evolved properties, as the strain was adapted with the medium. The composition was shown as follow: K_2_HPO_4_ 3.88 g/l, NaH_2_PO_4_ 1.63 g/l, (NH_4_)_2_SO_4_ 2.00 g/l, MgCl_2_.6H_2_O 0.1 g/l, Ethylenediaminetetraacetic acid (EDTA) 10 mg/l, ZnSO_4_.7H_2_O 2 mg/l, CaCl_2_.2H_2_O, 1 mg/l, FeSO_4_.7H_2_O 5 mg/l, Na_2_MoO_4_.2H_2_O 0.2 mg/l, CuSO_4_.5H_2_O 0.2 mg/l, CoCl_2_.6H_2_O 0.4 mg/l, MnCl_2_.2H_2_O·1 mg/l. This medium was supplemented with different concentrations of ferulate (15–125 mM for ALE, 15–180 mM for growth comparison, 100 mM for the transformation of adapted ADP1 and 110 mM for 1-undecene production). The medium was also supplemented with different concentrations of vanillate (15 mM to 180 mM) for growth comparison. To prepare the stock solution (200 mM) of ferulate, proper amount of ferulic acid (Sigma-Aldrich) was added to deionized water, after which equimolar amount or slight excess of NaOH was slowly added while stirring until ferulic acid was completely dissolved. Vanillate stock solution was prepared in the same way. For solid medium, 15 g/l agar was added. Chloramphenicol (25 μg/ml) was added when needed.

### Adaptive laboratory evolution

The ferulate-tolerant strains were evolved by a short-term serial passage in mineral salts medium supplemented with ferulate as sole carbon source. Wild type *A. baylyi* ADP1 was first cultivated on solid mineral salts medium containing 15 mM ferulate. Single colony was selected and precultivated in Erlenmeyer flask (100 ml) containing 10 ml medium supplemented with 45 mM ferulate at 30 °C, 300 rpm. When reaching log phase, the cells were cryopreserved as parental strain and the culture was passaged to two Erlenmeyer flasks (100 ml) containing 10 ml of the same medium as in the precultivation. The resulted two populations were evolved in parallel. The concentration of ferulate was initially 45 mM and gradually increased during the evolution to maintain the selection pressure. Cells were passaged to fresh medium before entering into stationary phase to avoid unwanted mutation. Before each passage, the OD of the culture was measured. The amount of inoculum was adjusted daily to make the initial OD of each cultivation between 0.03 and 0.1. The cells were cryopreserved at − 80 °C every two passages. The evolution went on for 2 months and more than 61 transfers were performed, corresponding to more than 350 generations. After the evolution, single colonies from both populations were screened out on plates. The strain with the best growth in ferulate, designated as adapted ADP1, was further compared with wild type ADP1 (the parental strain).

### Comparison of growth between wild type and adapted ADP1

Wild type and adapted ADP1 were precultivated in 5 ml mineral salts medium supplemented with 15 mM ferulate at 30 °C and 300 rpm. After 24 h, the cells from both precultures were transferred to 96-well plate containing 200 μl mineral salts medium supplemented with different concentrations of ferulate (15 mM to 180 mM) respectively. Each cultivation was performed in triplicate. The cells were then incubated in Spark multimode microplate reader (Tecan, Switzerland) at 30 °C for 72 h. Shaking was performed for 5 min twice an hour with a frequency of 54 rpm. The optical density (OD) at 600 nm was measured every hour.

Wild type and adapted ADP1 were also compared regarding the growth in vanillate, the metabolite derived from ferulate in the aromatic catabolizing pathway. The same processes as above were used for the comparison but vanillate was used instead of ferulate.

### Plasmid construction and transformation

Plasmid construction was carried out using *E. coli* XL-1 Blue as host. The reagents for PCR, digestion and ligation were provided by Thermo Scientific (USA) and used according to provider’s instruction. The primers used in this study are listed in Table 1.

**Table 1:** List of primers used in this study

Name	Description	Oligo sequence (5–3′)
ab156	chnR/pChnB, PstI	GTTTCTTCCTGCAGCGGCCGCTACTAGTAGATTACGACATGTGAATTTATTCAAAATCTGC
ab155	chnR/pChnB, EcoRI	GTTTCTTCGAATTCGCGGCCGCTTCTAGAGTCTAGGGCGGCGGATTTGTCC
ab152	pBAV1C rev.	TCATGAATCAAAGGACGCTATTG
ab151	pBAV1C for.	GTCAAATATTCATAAGAACCTTTGATATAATC
GFP fwd	GFP	TGGAATTCGCGGCCGCTTCTAGAGAAAGAGGAGAAATACTAGATGCGTAAAGGTGAAGAACTGTTCAC
GFP rev	GFP	TAATACTGCAGTTAAGCTACTAAAGCGTAGTTTTCGTCGTTTGCAGCAGGCCTTTTGTAGAGTTCATCCATGCCGTG
vs15_2 44	*undA*, PstI	TAATCTGCAGCGGCCGCTACTAGTATTATCAGCCCGCAGCCAAC
vs15_1 68	*undA*, XbaI	TAATGAATTCGCGGCCGCTTCTAGAGAAAGAGGAGAAATACTAGATGATTGACGCATTTGTTCGTATC
tl15	‘*tesA*, XbaI	TGGAATTCGCGGCCGCTTCTAGAGAAAGAGGAGAAATACTAGATGGCGGACACGTTATTGATTCTGGG
tl16	‘*tesA*, PstI	GTTTCTTCCTGCAGCGGCCGCTACTAGTATTATTATGAGTCATGATTTACTAAAGGCTGC

The plasmid pBAV1C-chnR/pChnB designated as pBAV1C-chn was constructed as follows. First, the plasmid pBAV1C-T5-GFP, which was constructed by Santala et al. [50], was amplified with the primers ab151 and ab152 to remove internal *Nde*I-site from the plasmid backbone. The PCR product was self-ligated and transformed to *E. coli* XL1-Blue strain. The obtained plasmid was named as pBAV1C-T5-GFP. Then, the fragment containing the regulator chnR and its cognate promoter pChnB was amplified from plasmid pSCM [a kind gift from Standard European Vector Architecture database (Spain)] [45], using primers ab155 and ab156. To obtain pBAV1C-chn, the amplified fragment was cloned to the pBAV1Cd plasmid backbone according to the BioBrick assembly standard 10 using EcoRI and PstI sites.

For the initial characterization of cyclohexanone-inducible promoter system (*ChnR*/P_chnB_) in *A. baylyi* ADP1, a plasmid pBAV1C-chn-GFP was constructed as follows. The gene fragment encoding monomeric and superfolder green fluorescent protein variant (GFP) was PCR amplified from the pSCM plasmid using BioBrick suffix and prefix primers, GFP fwd and GFP rev. The fragment was BioBrick-cloned to pBAV1C-chn plasmid to obtain pBAV1C-chn-GFP plasmid which was then transformed into *E. coli* XL-1 Blue. Verified pBAV1C-chn-GFP construct was transformed into *A. baylyi* ADP1 by natural transformation; Briefly, ADP1 was streaked on LA plate (containing 1% glucose). The cells were incubated at 30 °C overnight. On the second day, 0.5 μl of plasmid was dropped on the top of a single colony and the cells were incubated for another day. On the third day, the enlarged single colony were picked up and mixed with 50–100 μl LB medium. The mixture was spread onto LA plate (containing 1% glucose and 25 μg/ml chloramphenicol) and incubated at 30 °C until colonies appeared.

The gene *undA* was amplified with primer vs15_2 44 and vs15_1 68 using the genomic DNA of *Pseudomonas putida* KT2440 as template. The fragment was cloned to pBAV1C-chn with BioBrick assembly standard using restriction sites XbaI/SpeI and PstI. The resulting construct was designated as pBAV1C-chn -undA. The gene ‘*tesA* was amplified with tl15 and tl16 from the genome of E. coli MG1655 and cloned to pBAV1C-chn, resulting in pBAV1C-chn-*‘tesA*. The construct pBAV1C-chn-*undA* was further digested with SpeI and PstI and ligated with the previously digested *undA*, resulting in the construct pBAV1C-chn-*‘tesA*-*undA*. The former two plasmids contain only *undA* or ‘*tesA* respectively. The latter plasmid contains both ‘*tesA* and *undA.*

The transformation of *E. coli* was carried out using electroporation and the transformants was screened on LA plates containing 25 μg/ml chloramphenicol. *A. baylyi* ADP1 was transformed with the three constructed plasmids and the empty pBAV1C-chn. The adapted ADP1 was transformed with pBAV1C-chn-*‘tesA*-*undA*. The transformation was carried out as described by Metzgar et al. [12], with an exception that mineral salts medium containing 100 mM ferulate was used as the medium for the transformation. The constructs were verified by restriction analysis.

### Cultivation

For testing the functionality of (*ChnR*/P_chnB_), ADP1 carrying pBAV1C-chn-GFP plasmid was cultivated in minimal salts medium containing 1% glucose, 0.2% casein amino acid and 25 μg/ml chloramphenicol at 30 °C and 300 rpm. When the OD at wavelength 600 nm reached 0.5–1, 1 mM cyclohexanone was added in the culture. Cultivation without the addition of cyclohexanone was used as reference. The cultivation was performed in duplicate. Samples were taken for OD and fluorescence measurement after 3 h of cultivation. For fluorescence measurement, appropriate dilution was made to ensure that samples contained the same amount of biomass. Fluorescence measurement was performed with Spark multimode microplate reader (Tecan, Switzerland) with wavelengths 485 nm (excitation) and 510 nm (emission) and the signal was proportioned to that of the non-induced cells.

The cultivation for plasmid selection was carried out with the three constructed strains, ADP1 *undA*, ADP1 ‘*tesA*, and ADP1 ‘*tesA*-*undA*. ADP1 containing empty plasmid was used as control. Cultivation was performed in duplicate. Cells were precultivated in 5 ml LB medium containing 0.4% glucose and 25 μg/ml chloramphenicol. After overnight cultivation, cells were transferred to 6 ml MA/9 medium containing 5% glucose, 0.2% casein amino acid and 25 μg/ml chloramphenicol and cultivated at 30 °C and 300 rpm. Initial OD was made to 0.05. Cells were induced with 1 mM cyclohexanone after 5 h of cultivation (OD around 1). After 1 h of induction, 5 ml culture was transferred to sealed headspace 20 ml vials (Agilent Technology, Germany) containing a stir bar and incubated at 25 °C and 300 rpm overnight.

The cultivation for 1-undecene production from ferulate was carried out with ADP1 ‘*tesA*-*undA* and adapted ADP1 ‘*tesA*-*undA*. Cells were precultivated in mineral salts medium containing 5 mM ferulate and 25 μg/ml chloramphenicol at 30 °C and 300 rpm. The chloramphenicol used in the cultivation was prepared with water to ensure that ferulate is the only carbon source and energy source. ADP1 ‘*tesA*-*undA* was precultivated for 48 h while adapted strain was precultivated for 24 h. After precultivation, cells were transferred to 110 ml flasks supplemented with 12 ml mineral salts medium containing 110 mM ferulate and 25 μg/ml chloramphenicol. The initial OD was 0.05. Cells were induced after 17 h of cultivation with 1 mM cyclohexanone. After 4.5 h of induction, 10 ml culture was taken from each flask and evenly distributed to two sealed headspace 20 ml vials containing stir bars (each vial contains 5 ml culture). The cells were then incubated at 25 °C and 300 rpm for 20 h.

### Analysis methods

The consumption of carbon sources was analyzed with high performance liquid chromatography. The samples were collected from the cultures and centrifuged at 20,000*g* for 5 min. The supernatant was taken and filtered with syringe filters (CHROMAFIL^®^ PET, PET-45/25, Macherey–Nagel, Germany). The filtered supernatant was diluted with sterile deionized pure water. The measurement of ferulate concentration was performed with Agilent Technology 1100 Series HPLC (UV/VIS system) equipped with G1313A auto sampler, G1322A degasser, G1311A pump and G1315A DAD. Rezex RFQ-Fast Acid H^+^ (8%) (Phenomenex) was used as the column and placed at 80 °C. Sulfuric acid (0.005 N) was used as the eluent with a pumping rate of 1 ml/min.

The detection of 1-undecene was performed with SPME-GCMS described by Rui et al. [28]. Briefly, after cultivation, the sealed headspace vials were placed in aluminum block at 25 °C. At the same time, the culture was stirred with a magnetic stirrer. An SPME fiber (d_f_ 30 μm, needle size 24 ga, polydimethylsioxane, Supelco, Sigma-Aldrich) was injected into the vials and held for 12.5 min for absorption. GC–MS analysis was performed with Agilent 6890 N GC system with 5975B inert XL MSD. The analytes were desorbed from the fiber in a splitless injector at 250 °C for 75 s and developed with helium as carrier gas with a flow rate of 1 ml/min. Temperature gradient was applied: 50 °C for 3 min, temperature ramped to 130 °C with a rate of 10 °C/min, then ramped to 300 °C with a rate of 30 °C/min, 300 °C for 5 min. The concentration of the extracellular 1-undecene from the culture was calculated based on standard curve. For the preparation of gas chromatography standard, 1-undecene was mixed with MA/9 medium in the headspace 20 ml vials and the total volume was 5 ml. The range of the standard concentration was from 10 µg/l to 1000 µg/l. The measurement for the standard was performed with the same method mentioned above. The standard curve was made by plotting the peak area from each concentration as a function of the concentration of each standard.

## Additional file

**Additional file 1.** Additional figures.

## Abbreviations

LDMs: lignin-derived molecules
ALE: adaptive laboratory evolution
FFAs: free fatty acids
CoA: coenzyme A
OD: optical density
ACP: acyl carrier protein
SPME: solid phase micro-extraction
GCMS: gas chromatography mass spectrometry
