# Supplementary material for: Synthetic metabolic pathway for the production of 1-alkenes from lignin-derived molecules
Source: Microb Cell Fact. 2019 Mar 11;18:48. doi: 10.1186/s12934-019-1097-x (PMC6410514; doi:10.1186/s12934-019-1097-x)
Supplement: Supplementary file 1 — Additional file 1. Additional figures. [file 12934_2019_1097_MOESM1_ESM.docx]

**Additional file 1**


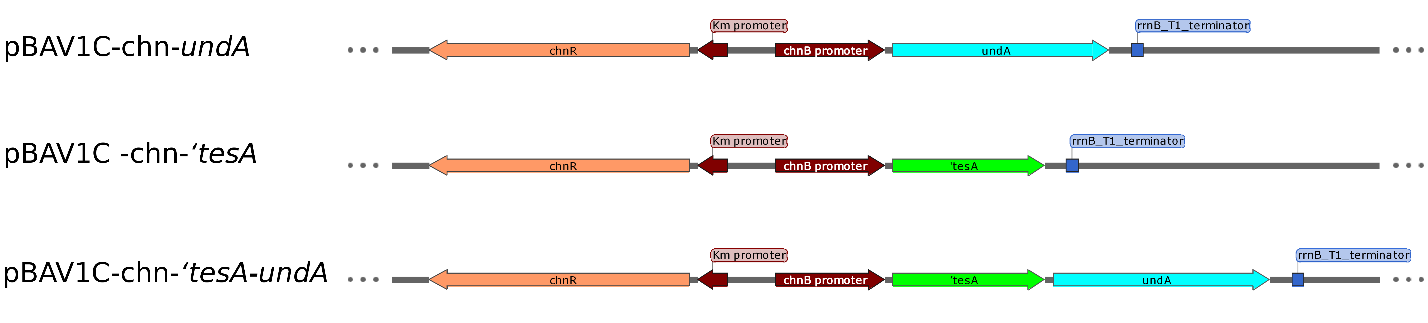


S1. A schematic of the constructed plasmids for 1-undecene production. Genes *undA* and ‘*tesA* are under control of a cyclohexanone-inducible promoter *ChnR*/P_chnB_ (indicated as chnB promoter in the figure). The regulator ChnR activates the transcription in the presence of cyclohexanone.


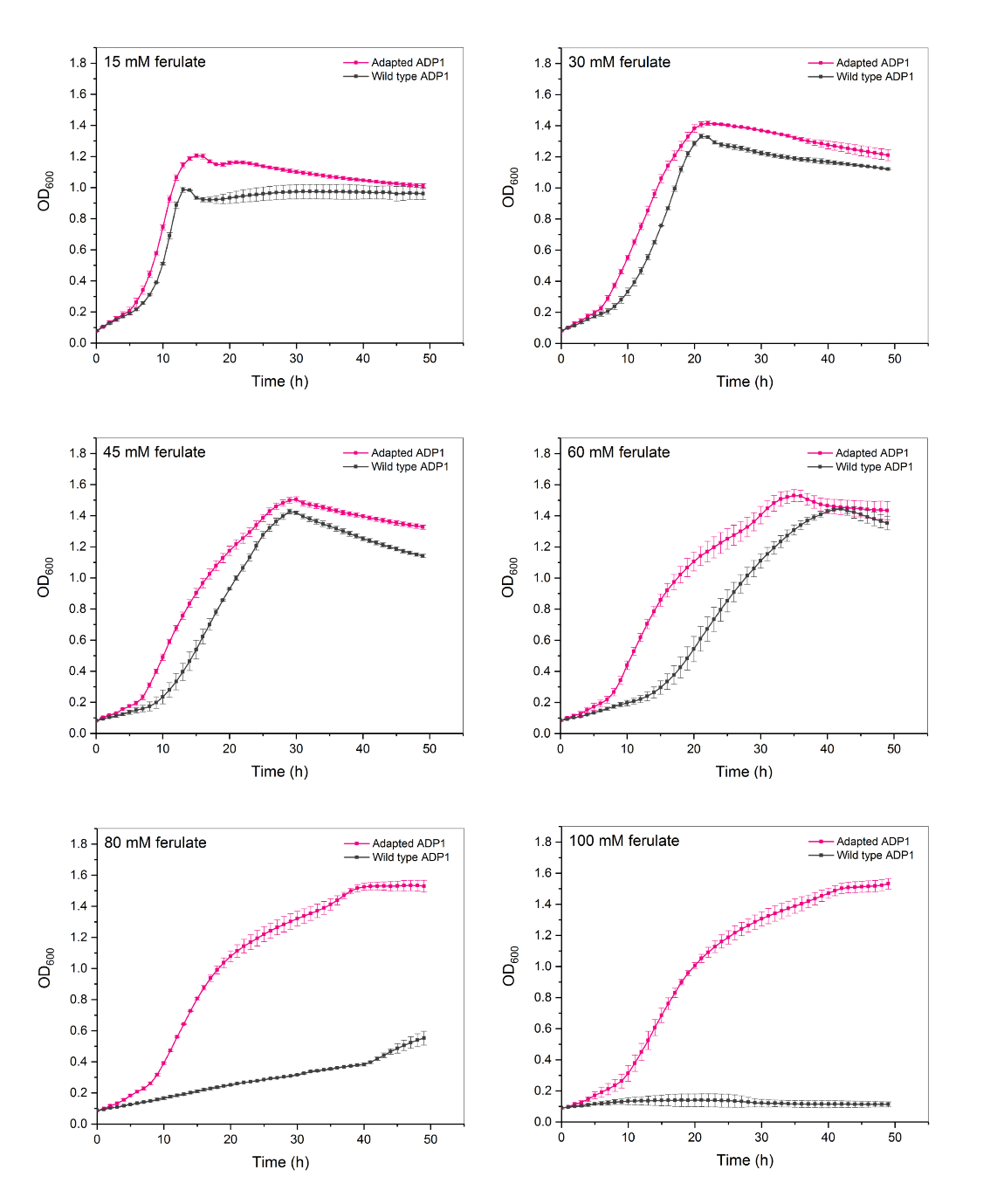


S2. Comparison of the growth between wild type and adapted ADP1 in different concentrations of ferulate. Both strains were cultivated in 96-well plate containing 200 μl mineral salts medium supplemented with different concentrations of ferulate. The cultivation was conducted in Spark multimode microplate reader (Tecan, Switzerland) at 30 °C. Shaking was performed for 5 min twice an hour with a frequency of 54 rpm and OD600 was measured every hour. The mean values and error bars (representing the standard deviations) from three parallel culture samples are shown.


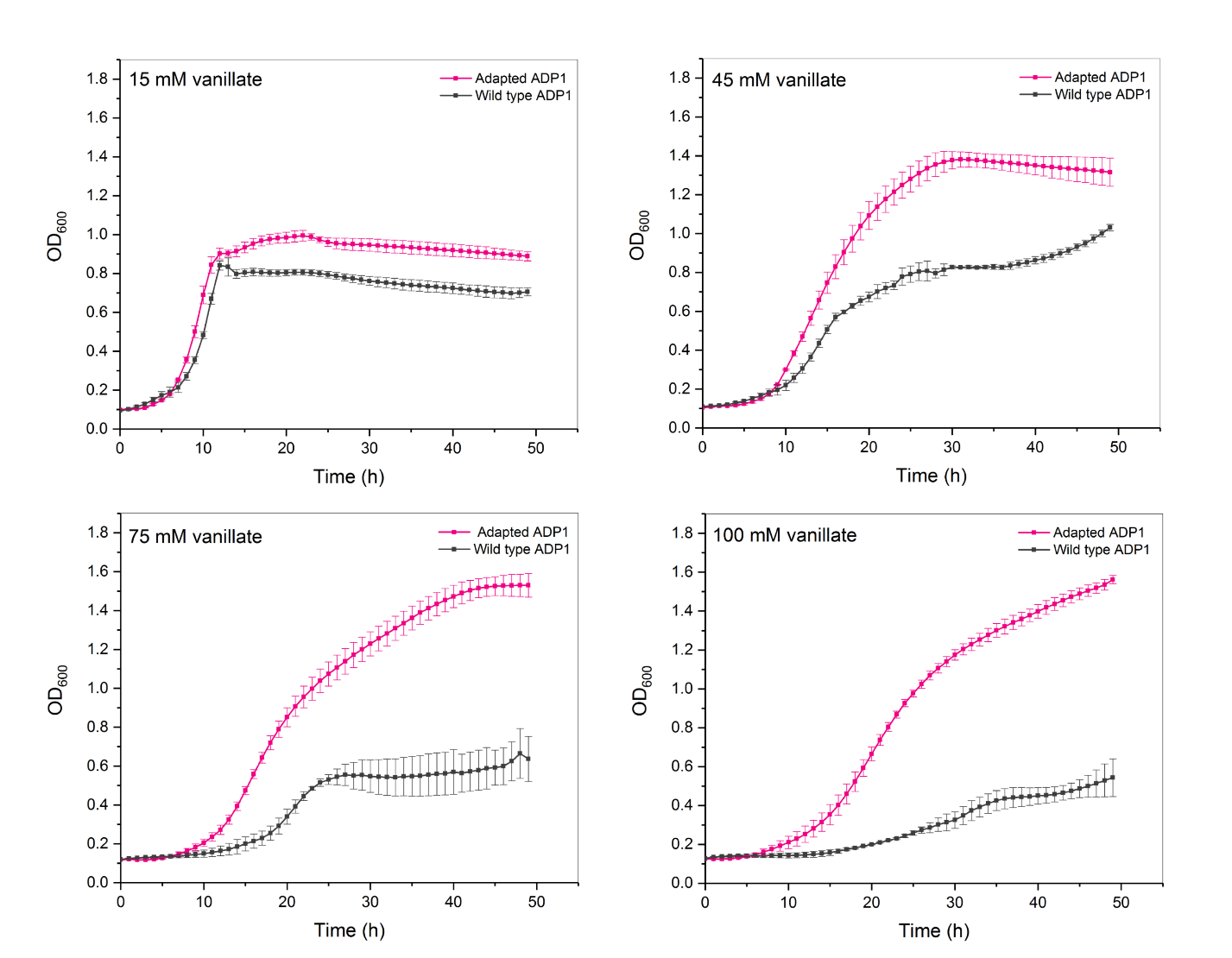


S3. Comparison of the growth between wild type and adapted ADP1 in different concentrations of vanillate. The cultivation was performed with the same experimental set-up and culture condition applied for the growth comparison in ferulate. The mean values and error bars (representing the standard deviations) from three parallel cultures are shown.


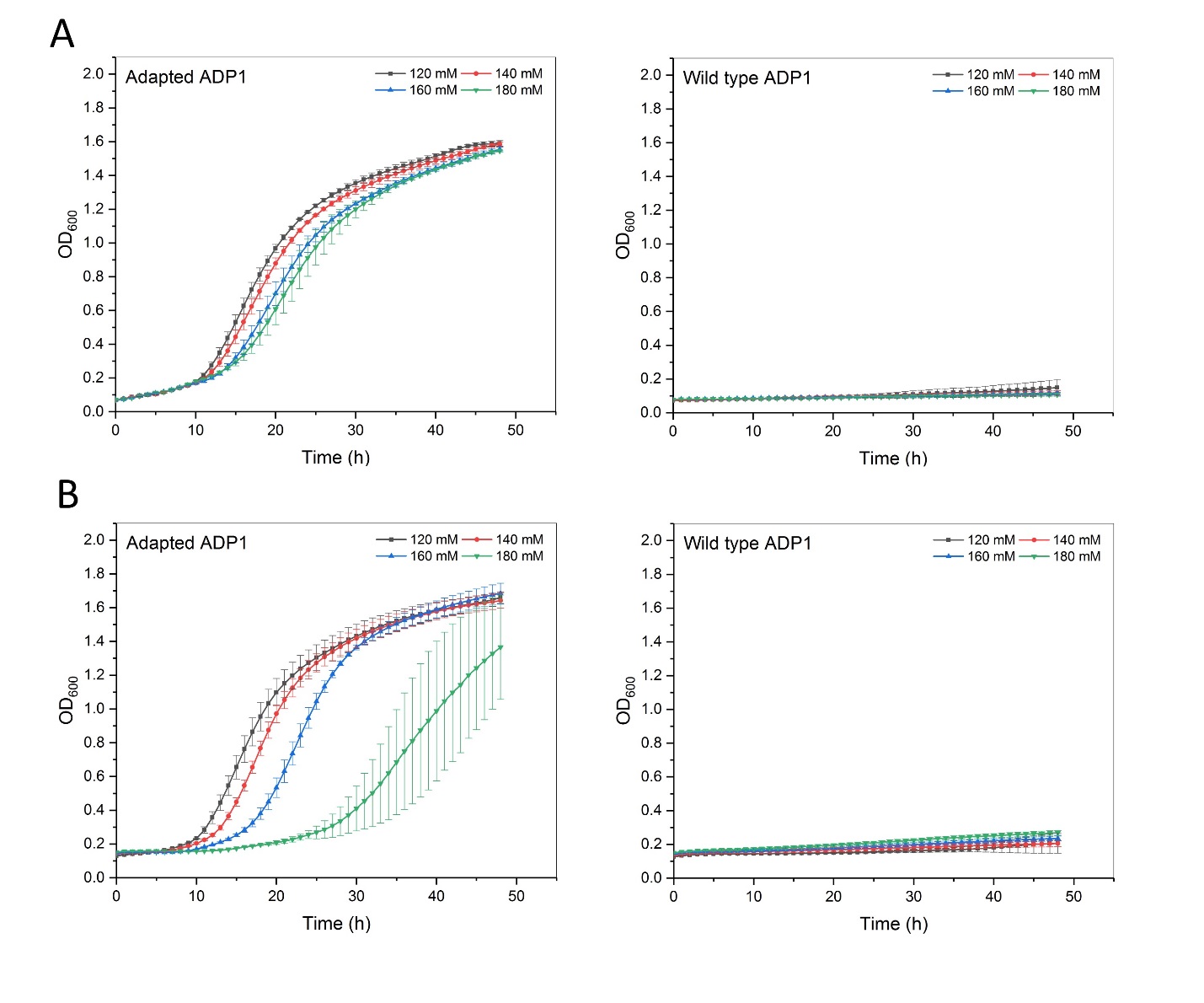


S4. The growth of adapted and wild-type ADP1 on ferulate (A) and vanillate (B) with higher concentrations (120 mM to 180 mM). Adapted and wild type ADP1 were cultured in mineral salts medium supplemented with different concentrations of ferulate and vanillate as sole carbon sources. For the cultivation of adapted ADP1 in 180 mM vanillate, the cells from one of the three parallel cultures did not grow, and thus the mean values and error bars (representing the standard deviations) from the other two parallel cultures are shown. For the other cultivations, the mean values and error bars from three parallel cultures are shown.

S5. The final OD of ADP1-empty plasmid (control), ADP1 *UndA*, ADP1 ‘*tesA*, and ADP1 ‘*tesA*-*undA* after the cultivation for 1-undecene production from glucose. Cells were first cultivated at 30 °C and 300 rpm. Inducer was added when OD was around 1. After 1 h of induction, the cultures were transferred to sealed vials and incubated at 25 °C and 300 rpm overnight. The mean values and error bars (representing the standard deviations) from two parallel culture samples are shown.


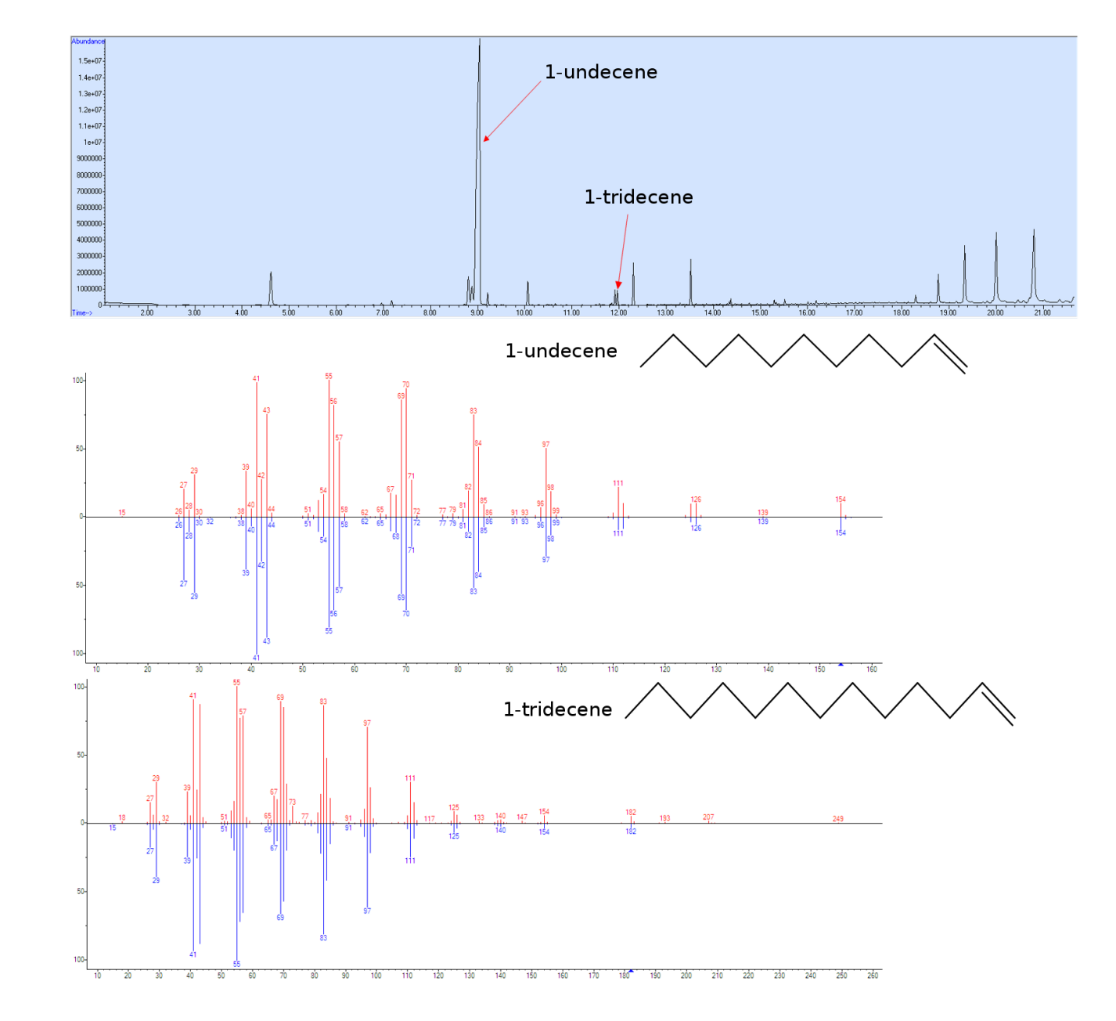


S6. GC-MS analysis of 1-undecene production from glucose. 1-Tridecene was also detected in the cultivation with ADP1 ‘*tesA*-*undA*. The reverse panel shows the reference standard of 1-undecene and 1-tridencene from NIST mass spectrometry data center.


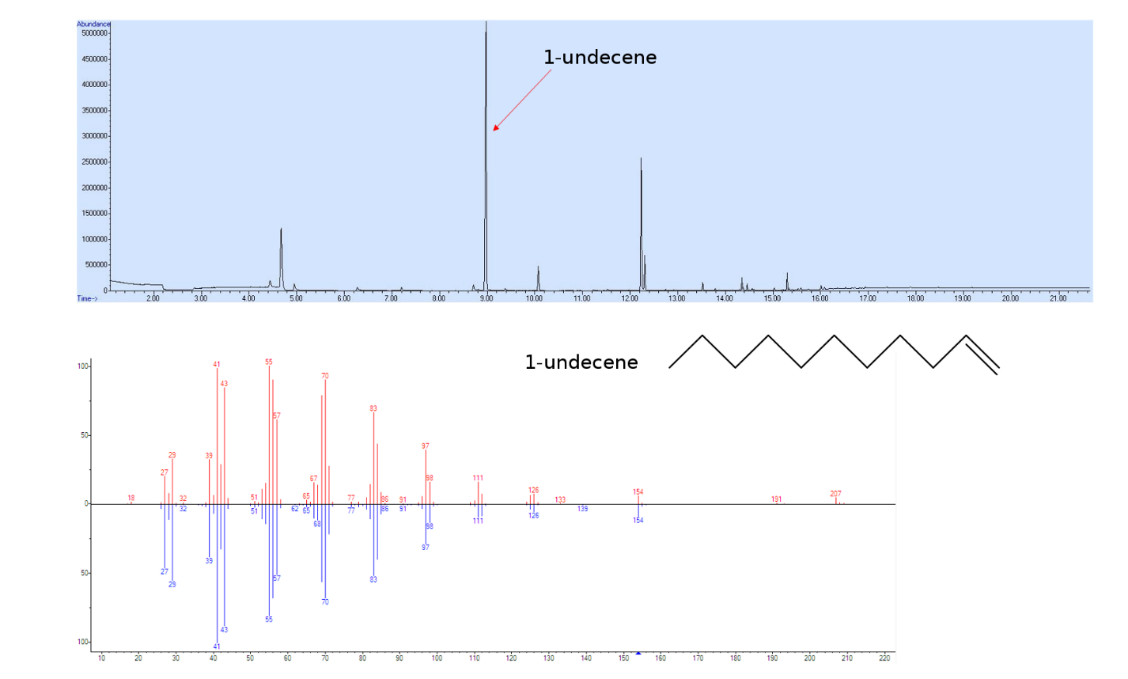


S7. GC-MS analysis of 1-undecene production from ferulate. The reverse panel shows the reference standard of 1-undecene from NIST mass spectrometry data center.
